# The Future of Emergency Medicine (EM) Sim Cases: A Modified Massive Online Needs Assessment

**DOI:** 10.7759/cureus.26799

**Published:** 2022-07-12

**Authors:** Anson Dinh, Teresa M Chan, Kyla Caners, Andrew K Hall, Andrew Petrosoniak, Tim Chaplin, Christopher Heyd, Jared B Baylis

**Affiliations:** 1 College of Medicine, University of Saskatchewan, Saskatoon, CAN; 2 Emergency Medicine, McMaster University, Hamilton, CAN; 3 Emergency Medicine, Queen's University, Kingston, CAN; 4 Emergency Medicine, University of Ottawa, Ottawa, CAN; 5 Emergency Medicine, University of Toronto, Toronto, CAN; 6 Emergency Medicine, The University of British Columbia, Kelowna, CAN

**Keywords:** digital scholarship, free open access medical education, education needs assessment, emergency medicine, simulation in medical education

## Abstract

Objective

Emergency Medicine (EM) Sim Cases was initially developed in 2015 as a free open-access simulation resource. To ensure the future of EM Sim Cases remains relevant and up to date, we performed a needs assessment to better define our audience and facilitate long-term goals.

Methods

We delivered a survey using a modified massive-online-needs-assessment methodology through an iterative process with simulation experts from the EM Simulation Educators Research Collaborative. We distributed the survey via email and Twitter and analyzed the data using descriptive statistics and thematic analysis.

Results

We obtained 106 responses. EM Sim Cases is commonly used by physicians primarily as an educational resource for postgraduate level trainees. Perceived needs included resuscitation, pediatrics, trauma, and toxicology content. Prompted needs included non-simulation-case educational resources, increased case database, and improved website organization.

Conclusions

Data collected from our needs assessment has defined our audience allowing us to design our long-term goals and strategies.

## Introduction

Emergency Medicine (EM) Sim Cases (https://emsimcases.com) is an online bank of simulation cases and educational content on a free open-access medical education (FOAM) platform. It was established to provide easy access to high-quality peer-reviewed simulation cases and to reduce the duplication of effort among simulation educators. EM Sim Cases currently serves over 10,000 visitors per month from over 200 countries.

To respond to the expanding and evolving profile of users and contributors and to inform new educational content moving forward, an intervention was needed to ensure that the needs of the EM Sim Cases audience were being met. As in most curricular or education innovation reforms, a formal needs assessment is critical to ensure the program in question continues to have its desired impact [1]. Using the Massive Online Needs Assessment (MONA) methodology [2], we set out to define both the reader’s perceived needs and the needs that readers were prompted to identify via an online survey. The aim of this process was to define and establish long-term goals for the site guided by participant needs. There are many FOAM resources in existence, however, there is a paucity of literature describing formal processes for determining content and ensuring the content is meeting the needs of participants.

This project was selected as a top-seven education innovation moderated poster presentation for the Canada Association of Emergency Physicians Annual Conference 2020 but could not be presented due to the COVID-19 pandemic.

## Materials and methods

Study setting and population

We made our survey available from October 15, 2019, to January 26, 2020. This project underwent review by the HiREB of McMaster University who waived full review, as it is considered program evaluation.

Participants were recruited via three avenues: 1) the EM Sim Cases email list, 2) a blog post on EM Sim Cases, and 3) via Twitter, with an infographic and link to the survey. We generated an email list of 407 participants from both subscribers to website updates and a pre-existing list of Canadian EM simulation experts [3]. One reminder was sent to this list. Twitter posts occurred three times through the EM Sim Cases Twitter account and twice through the email list of simulation experts.

Survey development

We developed the survey tool (Appendix 1) in keeping with established best practices in survey design [4]. This included an iterative process with simulation experts from the Canadian EM Simulation Educators Research Collaborative [5]. The survey included questions about user demographics (location, specialty, education, years of training, and level of website use) and the educational impact of EM Sim Cases as perceived by the respondent (how EM Sim Cases is used, for whom, and the level of involvement in local curricula). We piloted the survey with a registered nurse, two emergency physicians, and a medical student, all of whom were not otherwise involved in the study. This served to ensure understanding and evidence of content and face validity with modifications made accordingly.

Our survey was designed as a modification of the previously described the MONA methodology [2]. The original MONA method is well-suited to eliciting the needs of a diverse audience that exists in online communities. Specifically, our modified MONA contained:

*Phase 1:* Elicitation of perceived needs. During this phase, the reader’s perceived needs were assessed with a given list of topics and asked if they would like to see related content. In this project, we asked about simulation-related educational topics.

*Phase 2*: Defining prompted needs. During this section, our modified MONA used free text to elicit needs that the reader prompted themselves.

Originally, the MONA method contained a third phase wherein educators attempted to measure unperceived needs characterized by the following statement, “I do not know what I don’t know”, using methods like multiple-choice questions. This third phase is used to identify knowledge gaps [2] in curriculum development regarding a specific topic. While useful in teacher-centered methods of instruction, we deemed it would be inappropriate for our more general project, and therefore we did not include this phase.

Analysis and outcomes

The analysis excluded “item nonresponses” to capture constructive comments from respondents. Quantitative data from demographics, educational impact, and phase one of the modified MONA were analyzed in Microsoft Excel (Microsoft Corporation, Redmond, WA) using descriptive statistics.

Thematic analysis of the qualitative data from educational impact and phase two of the modified MONA was performed by two investigators (AD, JB), one of whom has extensive experience in simulation and another who was blinded to survey development. Authors independently analyzed and coded survey responses using an open coding technique [6]. They subsequently compared their results and agreed on a set of thematic codes that served as ‘suggestions’ to be improved upon. Data were recoded a second time and then sent to the author group for approval before manuscript production. We set our cut-offs at 20-40% for moderate priority and >40% for high priority to stratify our results to suit our curriculum development needs.

## Results

Demographics

We received 106 completed surveys from 18 countries, with the majority from Canada (n=41), the USA (n=27), and Australia (n=9). Email recruitment yielded 53 responses for a response rate of 13% while our blog post yielded 30 responses for a response rate of 26%. The remainder of the responses were obtained through Twitter, where a response rate calculation was not possible. Respondents consisted of physicians (n=59), nurses (n=12), and postgraduate trainees (n=10), with the majority specialized in emergency medicine (n=60). Other respondents (n=10) included paramedics, respiratory therapists, and medical students. Respondents most frequently reported six to 10 years of experience in their clinical career.

Educational impact

Notably, respondents reported that EM Sim Cases changed their clinical practice in that they added to their knowledge or provided a refresher (n=21) and overall positively impacted clinical practice (n=40). Many respondents noted specific impacts in free-form text responses (Table 1).

**Table 1:** "How has EM Sim Cases impacted your educational practice?"

Free-text Responses
“I find the cases helpful. It is quite a bit of work to make up ones own cases and its great to have a database for content.”
“Definitely came here looking for a sim template to communicate with my sim team through a shared language. Amazed at the detail and “like-minded” attention to high fidelity.”
“Has helped tremendously in re-establishing a simulation program that was left in shambles. Currently finishing up first semester of cases that couldn't have been orchestrated without help from the site.”
“Amazing resource for clinical scenarios that can be customized to meet the need of the learner.”
“I feel they make better pre-hospital providers. Practicing complex cases is helpful.”

The majority (n=75) used EM Sim Cases as a resource for simulation facilitation and/or exam preparation. Many respondents (n=57) used completed published cases and a minority (n=23) used our template for their own development of cases. Respondents (n=92) used the site as part of their simulation curriculum targeting postgraduate trainees (n=67), nurses (n=53), and medical students (n=46). Four respondents noted that 76-100% of their simulation curriculum was made up entirely of EM Sim Cases material. Cases were also used for the continued professional development of staff physicians (n=35).

Future directions

**Figure 1:**
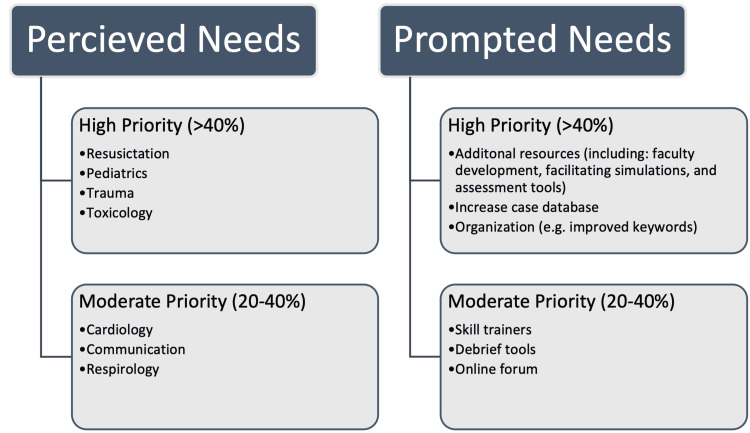
Breakdown of needs acquired through the MONA component of EM Sim Cases needs assessment survey MONA: Massive Online Needs Assessment; EM: emergency medicine

In phase one of our MONA, resuscitation, pediatrics, trauma, and toxicology cases were reported as high-priority perceived needs (Figure 1). Phase two of our MONA found faculty development, facilitating simulations, and assessment tools to be high-priority prompted needs for additional resources. Other high priorities were more cases and better website organization (e.g. improved searchable keywords). A notable request for more rural and disaster medicine cases was identified in our survey, however, this did not meet outcome percentage endorsement cut-offs.

## Discussion

Our data demonstrated that EM Sim Cases is frequently used to support educational curricula at the program and institutional levels. EM Sim Cases may serve to overcome some of the commonly reported barriers to simulation-based education, including lack of time and medical simulation expertise [7-8]. Specifically, a standardized template reduces the time required to start from scratch, an intimidating undertaking for those unfamiliar with simulation case design. Pre-written simulation cases reduce the work required by educators thereby simplifying the design and delivery of simulation scenarios. In addition, it may enhance trust in the simulation educator that the simulation session will go as intended since it has been previously trialed elsewhere. The utility of the site will continue to benefit from increased usage, much like Wikipedia has harnessed the wisdom of crowds.

Our MONA identified priorities that will improve EM Sim Cases as an educational resource. First, respondents identified perceived needs of priority case topics: resuscitation, pediatrics, trauma, and toxicology. These are in line with a recent survey of Canadian EM training programs [7] and previous reports of perceived deficiencies in residency education [9-10]. Second, respondents indicated that additional website content and improved organization would be beneficial. Proposed organizational improvements included an improved search function for cases (e.g., improved keywords) and a more appealing layout. To achieve these goals with a limited workforce, EM Sim Cases may need to adopt a post-publication peer review system incorporating both comments and expert feedback [11].

We identified a signal toward the need for more disaster medicine content that did not meet prespecified cutoffs. This result may have been augmented had the survey been completed during the coronavirus disease 2019 (COVID-19) global pandemic. In-situ simulation played a key role in many emergency departments to improve the safety of workers during the COVID-19 pandemic [12]. On Feb 18, 2020, EM Sim Cases published a high-acuity COVID-19 simulation case, which received significant interest with 18,265-page views and 7,543 case downloads over the span of four months. This heavily utilized case was downloaded and used across the globe for healthcare provider training and system improvement and serves as an example of how identified needs can be met in short order by an online community of practice such as EM Sim Cases.

Limitations

Our modified MONA has several limitations. Our response rate was low, attributed to our poor email response rate, which may lead to a selection bias in those who respond versus the typical user. Additionally, our respondents were primarily physicians, and future needs assessment surveys will need to reflect interdisciplinary education in a more robust manner.

## Conclusions

As EM Sim Cases continues to expand, the needs and expectations of its users continue to evolve. Re-evaluation was necessary to quantify and qualify these needs. Data gathered from our survey will assist to support the redesign of this educational resource and align our priorities with those of our users.

## Appendices

Appendix 1: the survey tool

1.     How often do you use EM Sim Cases? 

a.     Weekly 

b.     Monthly 

c.     Several times per year 

d.     Once per year 

e.     Never 

2.     Do you use EM Sim Cases solely for the simulation cases, solely for the other blog content, or for both? 

a.     Solely for other blog content

b.     Solely for the simulation cases 

c.     Both 

d.     Neither

3.     What do you use EM Sim Cases material for (e.g. education, in situ simulation, quality improvement, studying, assessment, etc)? 

4.     How much (if any) of your simulation curriculum is made up of EM Sim Cases material?

a.     None

b.     <25% 

c.     25-50%

d.     51-75% 

e.     >75% 

f.      Unsure

5.     How has EM Sim Cases impacted your educational practice (if at all)?

6.     How has EM Sim Cases impacted your clinical practice (if at all)? 

7.     We currently have the following categories of simulation cases. Of this list, please select the three that interest you the most? 

a.     Resuscitation 

b.     Pediatrics 

c.     Trauma 

d.     Toxicology 

e.     Respiratory

f.      OBGYN

g.     GI

h.     Neurology

i.       Endocrine

j.       Communication

k.     Cardiology

8.     What other resources do you use when sourcing simulation cases aside from EM Sim Cases? 

9.     When referring to sources other than EM Sim Cases, what do you like and/or dislike about those resources?

10.  Are there any simulation cases that you would like to see developed that have not yet been covered? 

11.  We are trying to expand our simulation-related blog content. In addition to content about simulation cases, what else would like to see?

12.  What could EM Sim Cases do to be more useful to you?

13.  In addition to your suggestions thus far, do you have any general suggestions for future directions of EM Sim Cases?

14.  What do you dislike about the website?

15.  What are your suggestions for how to improve the website?

16.  Have you ever submitted a case or a post for EM Sim Cases? Why or why not?

17.  What field do you come from?

a.     Staff physician

b.     Resident

c.     Medical student

d.     Respiratory therapist

e.     Physician assistant

f.      Simulation technologist

g.     Registered nurse

h.     Licensed practical nurse

i.       Nurse practitioner

j.       Primary care paramedic

k.     Advanced care paramedic

l.       Critical care paramedic

m.    Other

18.  How many years into your career are you?

a.     In training

b.     0-5 years

c.     6-10 years

d.     11-15 years 

e.     16-20 years

f.      21+ years

19.  If you are a physician, what specialty are you from (e.g. emergency medicine, family medicine, general surgery, etc)? 

20.  What is your training background in simulation-based education?

a.     Completed a simulation fellowship

b.     Masters degree with a focus on simulation-based education

c.     Completed a simulation debriefing course (e.g. the Harvard debriefing course)

d.     Informal training

21.  Are you a simulation leader/educator who is involved in simulation curriculum design and/or delivery? 

a.     Yes 

b.     No 

22.  If you answered yes to the previous question, which group of learners does this pertain to (check all that apply)?

a.     Residents

b.     Medical students

c.     Nursing

d.     Paramedicine

e.     Respiratory therapy

f.      Staff physician continuing professional development

g.     Physician assistants

23.  Where did you discover this survey? 

a.     Email

b.     EM Sim Cases blog post

c.     Twitter

24.  What city and country do you live in primarily?

## References

[1] Hall AK, Hagel C, Chan TM, Thoma B, Murnaghan A, Bhanji F (2018). The writer's guide to education scholarship in emergency medicine: education innovations (part 3). CJEM.

[2] Chan TM, Jo D, Shih AW (2018). The Massive Online Needs Assessment (MONA) to inform the development of an emergency haematology educational blog series. Perspect Med Educ.

[3] Kester-Greene N, Hall AK, Walsh CM (2019). Simulation curricular content in postgraduate emergency medicine: a multicentre Delphi study [Article in French]. CJEM.

[4] Kelley K, Clark B, Brown V, Sitzia J (2003). Good practice in the conduct and reporting of survey research. Int J Qual Health Care.

[5] Chaplin T, Thoma B, Petrosoniak A (2020). Simulation-based research in emergency medicine in Canada: priorities and perspectives. CJEM.

[6] Gibbs GR (2018). Analyzing Qualitative Data. https://www.perlego.com/book/3013539/analyzing-qualitative-data-pdf?utm_source=google&utm_medium=cpc&campaignid=15825112969&adgroupid=132780871355&gclid=Cj0KCQjwzLCVBhD3ARIsAPKYTcRsfnTe169LOsrtOruVlS61oXIFhbYbk6hNe7kWivUR87NKrjP0LJ0aAgHqEALw_wcB.

[7] Russell E, Hall AK, Hagel C, Petrosoniak A, Dagnone JD, Howes D (2018). Simulation in Canadian postgraduate emergency medicine training - a national survey. CJEM.

[8] Baschnegger H, Meyer O, Zech A, Urban B, Rall M, Breuer G, Prückner S (2017). Full-scale simulation in German medical schools and anesthesia residency programs: status quo [Article in German]. Anaesthesist.

[9] Bank I, Cheng A, McLeod P, Bhanji F (2015). Determining content for a simulation-based curriculum in pediatric emergency medicine: results from a national Delphi process. CJEM.

[10] Saqe-Rockoff A, Ciardiello AV, Schubert FD (2019). Low-fidelity, In-situ pediatric resuscitation simulation improves RN competence and self-efficacy. J Emerg Nurs.

[11] Thoma B, Chan T, Desouza N, Lin M (2015). Implementing peer review at an emergency medicine blog: bridging the gap between educators and clinical experts. CJEM.

[12] Chaplin T, McColl T, Petrosoniak A, Hall AK (2020). "Building the plane as you fly": simulation during the COVID-19 pandemic. CJEM.

